# Comparison of Long-Acting Injectable Antipsychotics With Oral Antipsychotics and Suicide and All-Cause Mortality in Patients With Newly Diagnosed Schizophrenia

**DOI:** 10.1001/jamanetworkopen.2021.8810

**Published:** 2021-05-11

**Authors:** Cheng-Yi Huang, Su-Chen Fang, Yu-Hsuan Joni Shao

**Affiliations:** 1 Department of Community Psychiatry, Bali Psychiatric Center, Ministry of Health and Welfare, New Taipei City, Taiwan; 2 Department of Nursing, Mackay Medical College, New Taipei City, Taiwan; 3 Graduate Institute of Biomedical Informatics, Taipei Medical University, Taipei, Taiwan; 4 Clinical Big Data Research Center, Taipei Medical University Hospital, Taipei, Taiwan

## Abstract

**Importance:**

Schizophrenia is generally considered to be among the most severe psychiatric disorders because of the excessive mortality associated with it. Research to find means to reduce this excessive mortality is warranted.

**Objective:**

To investigate associations of long-acting injectable antipsychotics (LAIs) with all-cause, natural-cause, and suicide mortality risks as well as the impacts of early use of LAIs in patients with newly diagnosed schizophrenia.

**Design, Setting, and Participants:**

This cohort study used data from the Taiwan National Health Insurance Research Database to construct a population-based cohort of patients with schizophrenia who received oral antipsychotics (OAPs) from January 1, 2002, to December 31, 2017. Within this cohort, the LAI group was defined as patients who switched to LAIs and were prescribed LAIs at least 4 times within 1 year. The LAI group was propensity matched 1:1 to patients who continued receiving OAPs of the same compounds. All patients were followed up until switching the antipsychotic administration route, death, or the end of the study (December 31, 2018), whichever occurred first. Data analysis was performed from January 2002 to December 2018.

**Main Outcomes and Measures:**

All-cause mortality, natural-cause mortality, suicide mortality, and suicide attempts.

**Results:**

In total, 2614 patients who switched to LAIs (median [interquartile range] {IQR} age, 30 [23-39] years) and 2614 who received OAPs (median [IQR] age, 30 [23-39] years) were included (1333 male patients [51.0%] in each group). During the 16-year follow-up period (median [IQR] follow-up of 14 [10-17] years), patients who switched to LAIs had lower risks of all-cause mortality (adjusted hazard ratio [aHR], 0.66; 95% CI, 0.54-0.81), natural-cause mortality (aHR, 0.63; 95% CI, 0.52-0.76), and suicide attempts (incidence rate ratio, 0.72; 95% CI, 0.55-0.93) compared with patients who received the corresponding OAPs. A 47% lower suicide mortality risk (aHR, 0.53; 95% CI, 0.30-0.92) was observed in patients who switched to LAIs within the first 2 years of OAP initiation.

**Conclusions and Relevance:**

These findings suggest that LAI use in patients with newly diagnosed schizophrenia is associated with decreased all-cause mortality and suicide risk. Furthermore, early treatment with LAIs within the first 2 years of OAP initiation was associated with a decrease in suicide mortality risk. Thus, LAI use in the early stage of treatment should be actively considered for patients with newly diagnosed schizophrenia.

## Key Points

**Question:**

Is use of long-acting injectable antipsychotics (LAIs) in patients with newly diagnosed schizophrenia associated with decreased mortality?

**Findings:**

This cohort study included 2614 patients with schizophrenia who switched to LAIs, and 2614 propensity-matched patients who continued receiving oral antipsychotics (OAPs) of the same compounds. Patients who switched to LAIs had lower mortality and fewer suicide attempts compared with patients who continued receiving the corresponding OAPs, and patients who switched to LAIs within the first 2 years of OAP initiation exhibited a 47% reduction in the risk of suicide mortality compared with patients who continued receiving OAPs.

**Meaning:**

These findings suggest that LAI use in patients with newly diagnosed schizophrenia should be encouraged because of its association with lower premature mortality.

## Teaser

This cohort study investigates the associations of long-acting injectable antipsychotics with all-cause, natural-cause, and suicide mortality risks in patients with newly diagnosed schizophrenia.

## Introduction

Schizophrenia is generally considered to be among the most severe psychiatric disorders because of the excessive mortality associated with it. It has consistently been associated with a life expectancy 10 to 25 years shorter than that of the general population.^1,2^ In a meta-analysis, Brown et al^3^ suggested that schizophrenia is associated with a large increase in suicide mortality and a moderately increased risk of natural-cause mortality. Therefore, research on practical methods for reducing these risks is highly warranted.

The lifetime risk of suicide in patients with schizophrenia is approximately 5%.^4^ The risk is highest during the early stage of the illness or the first episode.^5^ The suicide rate for patients with schizophrenia spectrum disorders is more than 20 times higher than that for the general population.^6^ Although suicide is a complex public health challenge, suicide is typically preventable with timely and evidence-based interventions. Risk factors for suicide in this population include being young, male, and highly educated. Illness-related risk factors include prior suicide attempts, depressive symptoms, active positive symptoms, and comorbid substance misuse.^4,7^ Effective treatment delivery and adherence are vital for suicide prevention in schizophrenia.^4,7^ Compared with oral antipsychotics (OAPs), long-acting injectable antipsychotics (LAIs) were reported to improve treatment adherence in patients with schizophrenia.^8,9^ However, studies of LAIs in protecting against suicide are limited; in particular, the association of LAIs with the course of the illness, if administered at an early phase, remains unclear.

The objective of the current study, therefore, was to investigate associations of LAIs and their equivalent OAPs on risks of all-cause, natural-cause, and suicide mortality in patients with newly diagnosed schizophrenia. We also explored whether patients benefited from the early use of LAIs.

## Methods

### Data Source

We conducted a population-based cohort study using data from the Taiwan National Health Insurance (NHI) Research Database (NHIRD) and the Taiwan Death Registry (TDR) database. The NHIRD is derived from Taiwan’s single-payer compulsory NHI program, which covers up to 99% of the 23 million people in Taiwan. The NHIRD includes all medical claims data on disease diagnoses, procedures, drug prescriptions, demographic characteristics, and enrollment profiles of all NHI beneficiaries.^10^ In addition, we linked the NHIRD to the TDR database to ascertain the vital status, date of death, and cause of death of patients. The accuracy of cause-of-death coding in the TDR database was previously validated.^11^

This study was reviewed and approved by the institutional review board of Taipei Medical University. A waiver of informed consent was granted because the patient information in the national claims data from the NHIRD was deidentified before analysis. All researchers signed an agreement guaranteeing patient confidentiality before using the database. This study is reported in accordance with the Strengthening the Reporting of Observational Studies in Epidemiology (STROBE) reporting guideline for cohort studies.^12^

### Study Population

#### Base Cohort

Our base cohort included patients with newly diagnosed schizophrenia (*International Classification of Diseases, Ninth Revision, Clinical Modification* [*ICD-9-CM*] code 295; *International Statistical Classification of Diseases and Related Health Problems, Tenth Revision *[*ICD-10*] codes F20 and F25) who initiated OAP treatment between January 1, 2002, and December 31, 2017 (170 261 patients). Patients were excluded on the basis of the following criteria: (1) aged less than 16 or greater than 65 years at cohort entry, (2) information regarding sex or age missing, (3) no antipsychotic prescription, and (4) LAI as the first antipsychotic prescription. Consequently, 138 240 patients were enrolled in the base cohort. The base cohort entry date was defined as the date of the first antipsychotic prescription.

#### Study Cohort

In our study, the LAI group was defined as patients in the base cohort who initiated an OAP prescription and then switched to LAIs during subsequent treatment and were prescribed LAIs at least 4 times within 1 year.^13,14^ In total, 35 182 patients (25%) switched to LAIs, and 13 826 (10%) were prescribed LAIs at least 4 times within 1 year. For each patient who received LAIs, we selected a propensity-matched reference patient from our base cohort who received an OAP of the same compound and continued receiving OAPs throughout. Using a prevalent new-user design,^15^ reference patients were selected from OAP initiators who spent the same duration of time in the base cohort as did exposed patients (defined as the period from the first prescription of OAPs to the first prescription of LAI) and continued receiving OAPs when exposed patients switched to LAIs. Accordingly, we randomly selected up to 1 reference patient in each exposure set who had matched OAPs of the same compounds received, age at cohort entry, sex, calendar year of cohort entry, psychiatric comorbidities 1 year before the index date, and psychiatric hospitalization (yes or no) 6 months before the index date with cases, using a time-conditioned propensity score. The index date was defined as the date of the first LAI prescription. All patients were followed up until switching the antipsychotic administration route, death, or the end of the study (December 31, 2018), whichever occurred first. The study flowchart and timeline are presented in eFigure 1 and eFigure 2 in the Supplement.

### Antipsychotic Exposure

Information on antipsychotics use was obtained from ambulatory and inpatient prescription claims data using the Anatomical Therapeutic Chemical Classification System code N05A (antipsychotics) but excluding N05AN (lithium).^16^ The antipsychotics and Anatomical Therapeutic Chemical codes used for the definition are shown in eTable 1 in the Supplement. Patients were stratified into LAI and OAP groups according to the study criteria. LAIs were further categorized into flupentixol, haloperidol, olanzapine, paliperidone, and risperidone to explore the associations of different drugs with the outcomes. To investigate the association of the early use of LAIs, we grouped patients using LAIs into those switching to LAIs within 2 years and those switching after 2 years, according to a literature review.^17,18,19,20^

### Outcomes

The study’s primary outcomes were all-cause mortality (*ICD-9-CM* codes 001-799 and E800-E999; *ICD-10* codes A00-Y98), natural-cause mortality (*ICD-9-CM* codes 001-799; *ICD-10* codes A00-R99), and suicide mortality (*ICD-9-CM* codes E950-E959; *ICD-10* codes X60-X84 and Y87), obtained by linking the NHIRD to the TDR database. Occurrences of all-cause and suicide deaths were determined from January 1, 2002, to December 31, 2018.^21^ The secondary outcome was suicide attempt occurrence, defined as emergency department (ED) visits and hospitalization with a diagnosis of suicide (*ICD-9-CM* codes E950-E959; *ICD-10* codes X60-X84).^22^

### Covariates

Covariates included age at cohort entry, sex, calendar year of cohort entry, psychiatric comorbidities 1 year before the index date, psychiatric hospitalization (yes or no), Charlson Comorbidity Index (CCI) score^23^ within 1 year before the index date, history of suicide attempts, and number of psychiatric ED visits within 6 months before the index date. The CCI score is associated with mortality and is a useful tool to measure the comorbid disease status in research including psychiatry.^1^ *ICD-10* and *ICD-9-CM* diagnosis codes for psychiatric and CCI scores are listed in eTable 2 in the Supplement.

### Statistical Analysis

Descriptive statistics were used to summarize patient characteristics in the LAI and OAP groups. Potential imbalances among covariates after matching were assessed using standardized mean differences. All-cause, natural-cause, and suicide mortalities per 100 000 person-years were independently estimated for the LAI and OAP groups. Conditional Cox regressions were used to estimate the risk of death.^24^ The Fine and Gray^25^ method was adapted to estimate the hazard of natural-cause and suicide deaths, considering competing risks from other causes of death. All Cox models included the CCI score within 1 year before the index date, history of suicide attempts, and number of psychiatric ED visits within 6 months before the index date in the model for adjustment. We used a Kolmogorov-type supremum test based on 1000 simulated residual patterns to test the validity of the proportional hazards assumption.^26,27^ The Cox model met the proportional hazards assumption (*P* > .05). Occurrences of suicide attempts were estimated on the basis of a negative binomial regression model by counting events and person-times of follow-ups. Significance was set at 2-tailed *P* < .05. All statistical analyses were performed using SAS statistical software version 9.4 (SAS Institute) and R statistical software version 4.0.0 (R Project for Statistical Computing). Data analysis was performed from January 2002 to December 2018.

## Results

### Patient Characteristics

The database extracted for the current analysis comprised 5228 patients with newly diagnosed schizophrenia (median [interquartile range] {IQR} age, 30 [23-39] years) who met the inclusion criteria (eFigure 1 in the Supplement). Further demographic and clinical characteristics of these patients are detailed in Table 1. Among them, 2614 patients who switched to LAIs (median [IQR] age, 30 [23-39] years) were compared with 2614 propensity-matched OAP (mean [IQR] age, 30 [23-39] years) patients in our study (1333 male patients [51.0%] in each group). The mean (SD) interval from the first LAI prescription to the fourth LAI prescription was 109 (69.3) days. Compared with the OAP group, the LAI group was more likely to have a history of suicide attempts and psychiatric ED visits 6 months before the index date, which implied that the LAI group consisted of patients with clinically more-severe disease (Table 1). The covariates mentioned were included in the matching and were evenly distributed between the 2 groups, except for the CCI score, history of suicide attempts, and number of psychiatric ED visits. Thus, these 3 covariates were not included in the matching.

**Table 1.** Baseline Characteristics of Patients Who Switched to LAIs and Their Matched Controls Who Continued Receiving OAPs of the Same Compounds

Characteristic	Patients, No. (%) (N = 5228)		SD^a^	*P* value
	LAIs (n = 2614)	OAPs (n = 2614)		
Demographic characteristics				
Age at first antipsychotics, median (IQR), y	30 (23-39)	30 (23-39)	0.00	NA
Age group, y				
16-35	1696 (64.9)	1691 (64.7)	0.01	NA
36-65	918 (35.1)	923 (35.3)	0.01	
Sex				
Male	1333 (51.0)	1333 (51.0)	0.00	NA
Female	1281 (49.0)	1281 (49.0)	0.00	
Duration from first antipsychotic to index date, median (IQR), mo	57 (20-105)	57 (20-105)	0.00	NA
Type of antipsychotic				
Haloperidol	455 (17.4)	455 (17.4)	0.00	NA
Flupentixol	619 (23.7)	619 (23.7)	0.00	
Olanzapine	152 (5.8)	152 (5.8)	0.00	
Risperidone	987 (37.8)	987 (37.8)	0.00	
Paliperidone	401 (15.3)	401 (15.3)	0.00	
Disease severity measured 1 y before the index date				
Psychiatric comorbidities				
Depression	477 (17.1)	477 (17.1)	0.00	NA
Anxiety disorder	520 (19.9)	520 (19.9)	0.00	
Bipolar disorder	493 (18.9)	493 (18.9)	0.00	
Substance use disorder	139 (5.3)	139 (5.3)	0.00	
Charlson Comorbidity Index score				
0	2435 (93.2)	2434 (93.1)		.47^b^
1	123 (4.7)	117 (4.5)		
2	39 (1.5)	36 (1.4)		
≥3	17 (0.7)	27 (1.0)		
Disease severity measured 6 mo before the index date				
Psychiatric hospitalization				
No	2076 (79.4)	2076 (79.4)	0.00	NA
Yes	538 (20.6)	538 (20.6)	0.00	
History of suicide attempts				
No	2556 (97.8)	2584 (98.9)		<.001^b^
Yes	58 (2.2)	30 (1.2)		
Psychiatric emergency department visits, No.				
0	2039 (78.0)	2235 (85.5)		<.001^b^
1	388 (14.8)	247 (9.5)		
≥2	187 (7.2)	132 (5.0)		

Abbreviations: IQR, interquartile range; LAI, long-acting injectable antipsychotic; NA, not applicable; OAP, oral antipsychotic.

^a^ SD = |*P*1 − *P*2|/[*P*1(1 − *P*1) + *P*2(1 − *P*2)/2]^0.5^. They are the same for all categorical variables with 2 levels. Charlson Comorbidity Index score, history of suicide attempts, and number of psychiatric emergency department visits were not included in matching.

^b^ The *P* value was generated using a χ^2^ test.

### All-Cause, Natural-Cause, and Suicide Mortality

During the follow-up period (median [IQR], 14 [10-17] years), 235 patients in the LAI group died and 287 patients in the OAP group died. All-cause mortality rates were 66 and 90 deaths per 100 000 person-years in the LAI and OAP groups, respectively (Table 2). The multivariate Cox model revealed that the LAI group had a 34% lower risk for all-cause mortality (adjusted hazard ratio [aHR], 0.66; 95% CI, 0.54-0.81) and a 37% lower risk of natural-cause mortality (aHR, 0.63; 95% CI, 0.52-0.76) compared with the OAP group after adjusting for the covariates. The median (IQR) follow-up time of suicide was 8 (5-11) years. The LAI group exhibited lower suicide mortality than the OAP group (13 vs 17 deaths per 100 000 person-years), but the risk estimate was not significant (aHR, 0.80; 95% CI, 0.58-1.11).

**Table 2.** All-Cause, Natural, and Suicide Mortality Risks in 5228 Patients With Schizophrenia Who Switched to LAIs Compared With Their OAP Using Counterparts

Mortality	Deaths, No.	Person-y, No.	Mortality rate, deaths/100 000 person-y (95% CI)	Cox regression model		Fine-Gray model	
				HR (95% CI)	aHR (95% CI)^a^	HR (95% CI)	aHR (95% CI)^a^
All cause							
LAIs	235	35 084	66 (58-75)	0.67 (0.55-0.81)	0.66 (0.54-0.81)	NA	NA
OAPs	287	31 643	90 (80-101)	1 [Reference]	1 [Reference]	NA	NA
Natural cause							
LAIs	153	35 084	43 (36-50)	0.62 (0.49-0.78)	0.62 (0.48-0.81)	0.63 (0.54-0.75)	0.63 (0.52-0.76)
OAPs	194	31 643	61 (52-70)	1 [Reference]	1 [Reference]	1 [Reference]	1 [Reference]
Suicide							
LAIs	48	35 084	13 (9-17)	0.91 (0.59-1.40)	0.87 (0.55-1.38)	0.87 (0.65-1.15)	0.80 (0.58-1.11)
OAPs	55	31 643	17 (13-22)	1 [Reference]	1 [Reference]	1 [Reference]	1 [Reference]

Abbreviations: aHR, adjusted hazard ratio; LAI, long-acting injectable; NA, not applicable; OAP, oral antipsychotic.

^a^ The aHR was derived from the Cox hazard model adjusted for the Charlson Comorbidity Index score within 1 year before the index date, suicide attempts, and the number of psychiatric emergency department visits within 6 months before the index date. The Fine and Gray method considered competing risks from other causes of death.

### Suicide Attempts

During the follow-up period, patients who switched to LAIs had fewer hospital visits for suicide attempts than their matched OAP controls (3.5 vs 4.2 visits) (Table 3). The negative binomial regression results indicated that patients who switched to LAIs exhibited a 28% lower risk of suicide attempts than patients who received only OAPs (incidence rate ratio, 0.72; 95% CI, 0.55-0.93).

**Table 3.** Risk of Suicide Attempts in the LAI and OAP Groups

Group	Patients, No.	Suicide attempts, mean (SD), No.	IRR (95% CI)^a^	*P* value
LAI	264	3.5 (6.5)	0.72 (0.55-0.93)	.01
OAP	263	4.2 (8.19)	1 [Reference]	

Abbreviations: IRR, incidence rate ratio; LAI, long-acting injectable antipsychotic; OAP, oral antipsychotic.

^a^ The IRR was estimated using a negative binomial regression model adjusted for the Charlson Comorbidity Index score within 1 year before the index date, suicide attempts, and the number of psychiatric emergency department visits within 6 months before the index date.

### Benefit of Early Use of LAIs

We examined the risk of mortality associated with the early use of LAIs (ie, within 2 years) compared with the matched OAP group (Table 4). In our population, 742 patients switched to LAIs within the first 2 years of OAP initiation, and 1872 switched to LAIs more than 2 years after OAP initiation. Patients who switched to LAIs within the first 2 years of OAP initiation had a 47% decreased risk in suicide mortality compared with those who continued receiving OAPs (aHR, 0.53; 95% CI, 0.30-0.92). The benefit of LAIs for protecting against suicide mortality was not observed in patients who switched to LAIs more than 2 years after OAP initiation (aHR, 1.03; 95% CI, 0.70-1.52). Regarding all-cause mortality, patients who switched to LAIs within 2 years had lower all-cause mortality than the OAP group (114 vs 129 deaths per 100 000 person-years), but the risk estimate did not reach statistical significance (aHR, 0.93; 95% CI, 0.67-1.30).

**Table 4.** Risk of Suicide Mortality in 5228 Patients With Schizophrenia Who Switched to LAIs Within 2 Years of OAP Initiation or More Than 2 Years After OAP Initiation Compared with Their Corresponding Counterparts

Group	Deaths, No.	Person-y, No.	Mortality rate, deaths/100 000 person-y (95% CI)	Cox regression model		Fine-Gray model	
				HR (95% CI)	aHR (95% CI)^a^	HR (95% CI)	aHR (95% CI)^a^
Switched to LAIs ≤2 y after initiating OAPs^b^							
All-cause mortality							
LAIs	94	8233	114 (93-139)	0.89 (0.65-1.22)	0.93 (0.67-1.30)	NA	NA
OAPs	92	7109	129 (105-158)	1 [Reference]	1 [Reference]	NA	NA
Natural-cause mortality							
LAIs	67	8233	81 (63-102)	1.04 (0.70-1.56)	1.25 (0.80-1.95)	1.06 (0.81-1.39)	1.22 (0.88-1.69)
OAPs	57	7109	80 (61-103)	1 [Reference]	1 [Reference]	1 [Reference]	1 [Reference]
Suicide							
LAIs	12	8233	14 (7-24)	0.61 (0.28-1.29)	0.59 (0.27-1.29)	0.55 (0.33-0.92)	0.53 (0.30-0.92)
OAPs	22	7109	30 (19-45)	1 [Reference]	1 [Reference]	1 [Reference]	1 [Reference]
Switched to LAIs >2 y after initiating OAPs^c^							
All-cause mortality							
LAIs	141	26 851	52 (44-61)	0.58 (0.46-0.74)	0.54 (0.41-0.70)	NA	NA
OAPs	195	24 534	79 (68-90)	1 [Reference]	1 [Reference]	NA	NA
Natural-cause mortality							
LAIs	86	26 851	32 (25-39)	0.47 (0.35-0.64)	0.41 (0.29-0.60)	0.49 (0.39-0.61)	0.42 (0.31-0.55)
OAPs	137	24 534	55 (46-65)	1 [Reference]	1 [Reference]	1 [Reference]	1 [Reference]
Suicide							
LAIs	36	26 851	13 (9-18)	1.11 (0.66-1.89)	1.10 (0.61-1.97)	1.1 (0.78-1.56)	1.03 (0.70-1.52)
OAPs	33	24 534	13 (9-18)	1 [Reference]	1 [Reference]	1 [Reference]	1 [Reference]

Abbreviations: aHR, adjusted hazard ratio; LAI, long-acting injectable antipsychotic; NA, not applicable; OAP, oral antipsychotic.

^a^ The aHR was derived using the Cox hazard model adjusted for the Charlson Comorbidity Index score within 1 year before the index date, suicide attempts, and the number of psychiatric emergency department visits within 6 months before the index date. The Fine and Gray method considered competing risks from other causes of death.

^b^ This group includes 1484 patients, 742 using LAIs and 742 using OAPs.

^c^ This group includes 3744 patients, 1872 using LAIs and 1872 using OAPs.

### Individual Antipsychotics

The effects of individual antipsychotics on mortality were also examined. The results indicated that the LAI forms of paliperidone (aHR, 0.30; 95% CI, 0.08-0.98), haloperidol (aHR, 0.61; 95% CI, 0.43-0.97), and risperidone (aHR, 0.71; 95% CI, 0.50-0.99) were associated with lower risks of all-cause mortality than were their corresponding OAPs (Figure).

**Figure.**
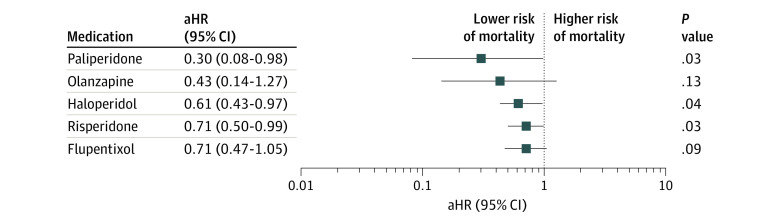
Adjusted Hazard Ratios (aHRs) Estimating the Risk of All-Cause Mortality Associated With Individual Long-Acting Injectable Antipsychotics Compared With Their Corresponding Oral Antipsychotics

## Discussion

In this study, the use of LAIs was associated with significantly lower risks of all- and natural-cause mortality and suicide attempts than was the use of OAPs after adjusting for covariates. With regard to the benefits of early use of LAIs, patients who switched to LAIs within the first 2 years of OAP initiation had a 47% decrease in the suicide mortality risk compared with those who continued receiving OAPs.

Here, the LAI group had a 34% lower all-cause and a 37% lower natural-cause mortality risk than did the OAP group. These results are in line with those of previous large observational cohort studies and meta-analyses of randomized clinical trials, in which receiving LAIs was associated with lower mortality than was receiving OAPs.^9,28,29^ Brown et al^30^ suggested that some of the excess mortality of schizophrenia could be lessened by reducing patient exposure to environmental risk factors and improving disease, mood disturbance, and psychosis management. Because LAIs were associated with lower risks of rehospitalization and antipsychotic treatment discontinuation than were OAPs,^9,14,29^ better outcomes in all-cause and natural-cause mortality might be attributable to improved medication adherence and less disease fluctuation and relapse.

With regard to associations of individual antipsychotics with mortality, the LAI forms of paliperidone, haloperidol, and risperidone were associated with lower risks of all-cause mortality compared with their corresponding OAPs. Notably, LAI flupentixol and LAI olanzapine were not associated with significant benefits for all-cause mortality. These findings are consistent with those of Taipale et al.^28^ However, studies further assessing the efficacy of individual antipsychotics against mortality are needed.

The lifetime risk of suicide in patients with schizophrenia is approximately 5%,^4^ and suicide is another leading contributor to excessive premature mortality in this population.^6,30^ The risk is highest among patients during the early stage of their illness or their first episode.^5^ Most suicide attempts occur within 2 years after the first episode of psychosis,^31,32^ and suicide-related mortality is higher among individuals recently diagnosed as having schizophrenia (ie, ≤5 years from diagnosis).^33^ The higher risk of suicide compared with later stages of schizophrenia might be explained by the critical period hypothesis, which states that the primary clinical and psychosocial deterioration associated with schizophrenia occurs within the first 5 years, the critical period.^34,35^ Within this period, biological, psychological, and psychosocial influences are developing and have maximum plasticity to adequate interventions. However, relapse is also common in this period because of low levels of insight and nonadherence to medication.^36^ Although suicide is a complex and multifactorial phenomenon, suicides are typically preventable with timely, evidence-based interventions. Hence, active early intervention and even the early application of LAIs to reduce risks of relapse, suicide attempts, and suicide mortality in the early stage have been proposed.^36,37,38,39^

Our findings suggest that LAI users had a lower suicide attempt risk and that patients who switched to LAIs within 2 years of OAPs treatment had decreased suicide mortality compared with those who continued receiving OAPs. These results are consistent with a previous study^38^ that revealed that the use of second-generation LAIs for recent-onset schizophrenia reduced suicidal ideation compared with chronic schizophrenia. However, we did not observe a decrease in suicide mortality among patients who switched to LAIs more than 2 years after OAP treatment. This might have been due to the neurological processes underlying schizophrenia having been ongoing for many years before the first episode.^40^ Therefore, with later LAI use, their protective effects might not be sufficient to compensate for the cognitive and psychosocial deterioration that occurs in the first few years of the illness.

Clinically, most psychiatrists use LAIs with a conservative attitiude,^41,42^ and the reasons for this attitude are generally not well supported by current scientific evidence.^41^ This attitude was also observed in our study; only 10% of our base cohort was prescribed LAIs at least 4 times within 1 year. Heres et al^43^ investigated factors associated with negative psychiatrist attitudes toward LAI use and identified 3 main ones: limited availability of different types of second-generation antipsychotic depot drugs, the frequent rejection of the depot by patients, and patient skepticism based on the lack of experiencing a relapse. Several other factors also influence psychiatrist practices, such as patient or psychiatrist perceptions, drug costs and insurance coverage, difficulty of adjusting LAI dosages in response to adverse effects, and the conservative position regarding LAI use in treating early-stage schizophrenia in the majority of guidelines.^14,44,45^

In our findings, the LAI group had significantly lower risks of all-cause and natural-cause mortality, and those who switched to LAIs in the early stage (≤2 years) had a 47% decreased risk in suicide mortality as well. Given that inadequate medication adherence is common among patients with newly diagnosed schizophrenia, more active consideration of LAIs in this stage for better long-term outcomes should be encouraged, particularly for those who have already exhibited poor adherence attitudes.^39,37,45,46,47,48^ Additional studies are also needed to clarify long-term adverse effects specific to LAIs use and identify specific psychotherapeutic and psychosocial interventions that may offer other benefits to patients in the early stage.

### Strengths and Limitations

This study’s major strength is its population-based design that used a prevalent new-user design to examine long-term mortality, for up to 16 years, as our primary outcome in patients with newly diagnosed schizophrenia. This design enabled head-to-head comparisons between LAIs and their corresponding OAPs by enrolling patients and considering the disease duration, age, sex, and psychiatric comorbidities at the baseline.

Several limitations should be addressed. First, all observational studies have residual confounding. However, we made our best effort to minimize this potential bias by stringently selecting our matched cohort using the duration of OAPs treatment, year of first antipsychotic treatment, and psychotic comorbidities at the baseline. Second, lifestyle factors, socioeconomic status, social support, and the personal preferences of psychiatrists were not included in the study because of the NHIRD’s limitations. Third, the CCI score, history of suicide attempts, and number of psychiatric ED visits were included in the Cox hazard model but not in the matching. Fourth, an immortal time bias may exist in cohort studies. In this study, the mean (SD) duration from the first LAI prescription to the fourth LAI prescription was 109 (69.3) days, and no participants died during this period. Thus, the likelihood of immortal time bias in our study was deemed to be low.

## Conclusions

Our findings suggest that the use of LAIs in patients with newly diagnosed schizophrenia was associated with significantly reduced risks of all-cause mortality and suicide attempts. Furthermore, early treatment with LAIs (within the first 2 years) was also associated with decreased suicide mortality risks. Thus, more active consideration of LAIs in the early stage of treatment should be encouraged.

## Notes

Supplement. **eFigure 1.** Flowchart Showing the Study Cohorts **eFigure 2.** Study Timeline **eTable 1.** ATC Codes Used to Identify Antipsychotics: Long-Acting Injectable Antipsychotics (LAIs) and Oral Antipsychotics (OAPs) **eTable 2.** *ICD-10* and *ICD-9 CM* Codes for Psychiatric and Charlson Comorbidities
