# Errors in Text, Figure, Tables, and Supplement

**DOI:** 10.1001/jamanetworkopen.2022.10829

**Published:** 2022-04-19

In the Original Investigation titled “Comparison of Long-Acting Injectable Antipsychotics With Oral Antipsychotics and Suicide and All-Cause Mortality in Patients With Newly Diagnosed Schizophrenia,”^1^ published May 11, 2021, there were multiple errors in the text, figure, tables, and supplement resulting from the misclassification of patients receiving rapid-acting intramuscular olanzapine as receiving long-acting olanzapine. To correct this error, the 152 patients receiving rapid-acting intramuscular olanzapine and the corresponding 152 patients receiving oral olanzapine were excluded, and data for risk of all-cause mortality, natural-cause mortality, suicide mortality, and suicide attempts were reanalyzed. This error did not affect the interpretation of the results, and the article has been corrected.^1^
