# Supplementary material for: Comparison of Long-Acting Injectable Antipsychotics With Oral Antipsychotics and Suicide and All-Cause Mortality in Patients With Newly Diagnosed Schizophrenia
Source: JAMA Netw Open. 2021 May 11;4(5):e218810. doi: 10.1001/jamanetworkopen.2021.8810 (PMC8114136; doi:10.1001/jamanetworkopen.2021.8810)
Supplement: Supplement. — eFigure 1. Flowchart Showing the Study Cohorts eFigure 2. Study Timeline eTable 1. ATC Codes Used to Identify Antipsychotics: Long-Acting Injectable Antipsychotics (LAIs) and Oral Antipsychotics (OAPs) eTable 2. ICD-10 and ICD-9 CM Codes for Psychiatric and Charlson Comorbidities [file jamanetwopen-e218810-s001.pdf]

Supplemental Online Content

Huang CY, Fang SC, Shao YHJ. Comparison of long-acting injectable antipsychotics with oral antipsychotics and suicide and all-cause mortality in patients with newly diagnosed schizophrenia. *JAMA Netw Open*. 2021;4(5):e218810. doi:10.1001/jamanetworkopen.2021.8810

eFigure 1. Flowchart Showing the Study Cohorts
eFigure 2. Study Timeline
eTable 1. ATC Codes Used to Identify Antipsychotics: Long-Acting Injectable Antipsychotics (LAIs) and Oral Antipsychotics (OAPs)
eTable 2. *ICD-10* and *ICD-9 CM* Codes for Psychiatric and Charlson Comorbidities

This supplemental material has been provided by the authors to give readers additional information about their work.

© 2021 Huang CY et al. *JAMA Network Open.*

eFigure 1. Flowchart Showing the Study Cohorts

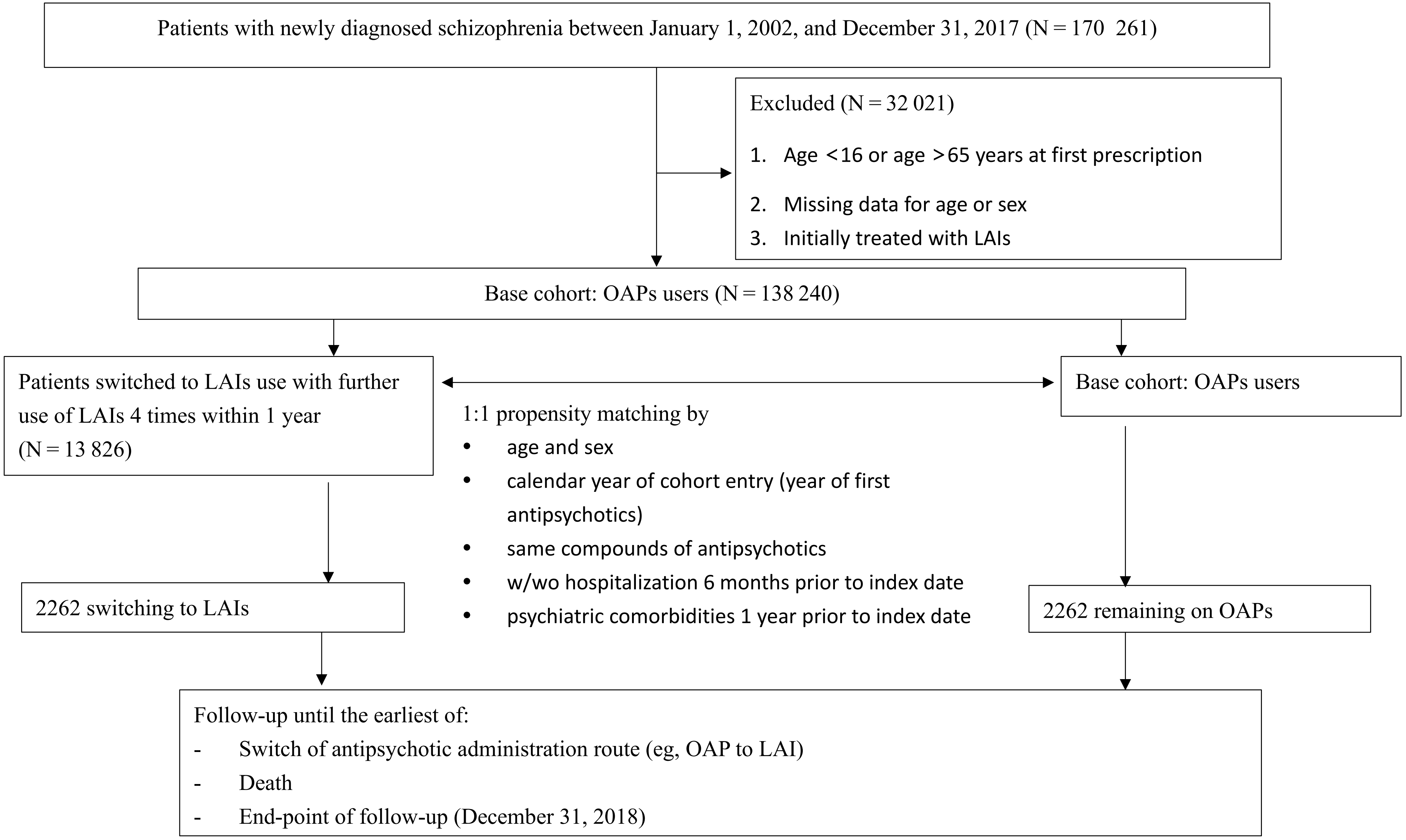

© 2021 Huang CY et al. *JAMA Network Open.*

eFigure 2: Study Timeline

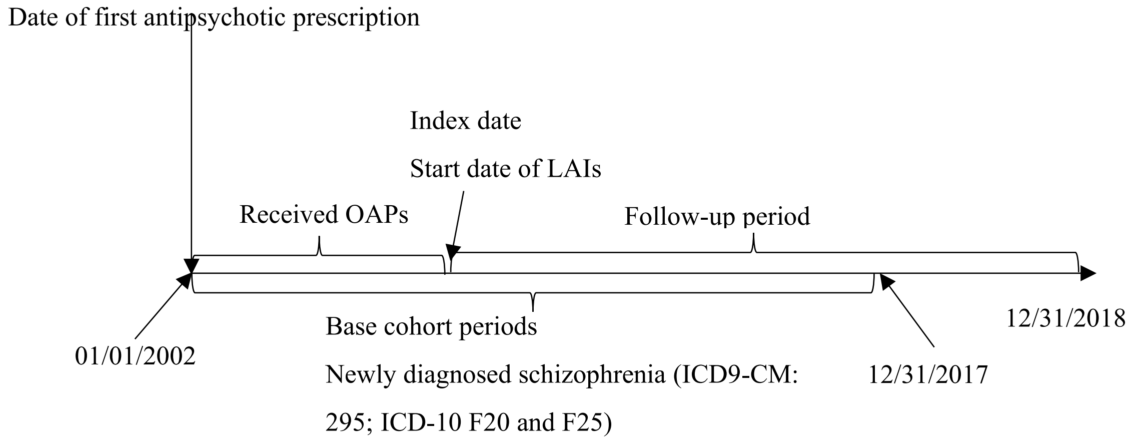

© 2021 Huang CY et al. *JAMA Network Open.*

eTable 1. ATC Codes Used to Identify Antipsychotics: Long-Acting Injectable Antipsychotics (LAIs) and Oral Antipsychotics (OAPs)

Antipsychotics and ATC code	Drug name
LAIs	
N05AA07	Clopenthixol
N05AB02	Haloperidol Zuclopenthixol Flupentixol
N05AD01	Fluphenazine
N05AF01	Olanzapine
N05AF05	Risperidone
N05AH03	Paliperidone
N05AX08	
N05AX13	
OAPs	
N05AA01	Chlorpromazine Levomepromazine Pimozide
N05AA02	Trifluoperazine
N05AA03	Chlorprothixene
N05AA05	Fluphenazine
N05AA07	Perphenazine
N05AB02	Prochlorperazine
N05AB03	Thioridazine
N05AB04	Pipotiazine
N05AC02	Haloperidol
N05AC04	Moperone
N05AD01	Ziprasidone
N05AD04	Flupentixol
N05AE04	Clopenthixol
N05AF01	Tiotixene
N05AF02	Zuclopenthixol
N05AF04	Penfluridol
N05AF05	Loxapine
N05AG03	Clozapine
N05AH01	Olanzapine
N05AH02	Quetiapine
N05AH03	Amisulpride
N05AH04	Sulpiride
N05AL05	Clothiapine
N05AL01	Aripiprazole
N05AX09	Risperidone

© 2021 Huang CY et al. *JAMA Network Open.*

N05AX12	Zotepine
N05AX08	Paliperidone
N05AX11	
N05AX13	

© 2021 Huang CY et al. *JAMA Network Open.*

eTable 2. *ICD-10* and *ICD-9 CM* Codes for Psychiatric and Charlson Comorbidities

Condition	ICD-9 CM codes	ICD-10 codes
Psychiatric comorbidities		
Depressive disorders	296.2x, 296.3x, 296.82, 300.4, 311	F32.x, F33.x, F34.1
Anxiety disorders	300.x except 300.4	F40.x~F42.x, F44.x, F45.x, F48.x, F68.11, F68.8, F99, R45.2
Bipolar disorder	296 excluding 296.2, 296.3, 296.82;	F30.x, F31.x, 39, F33.8, F34.8, F34.9
Substance use disorder	291.x, 292.x, 303.x, 304.x, 305.x, 357.5, 425.5, 535.3, 571.1~571.3	F10-F19, F55, G62.1, I42.6, K29.2, K70.9, K70.0~K70.3
Charlson comorbidities		
Acute myocardial infarction	410, 412	I21, I22, I252
Congestive heart failure	428	I50
Peripheral vascular disease	441, 4439, 7854, V434	I71, I790, I739, R02, Z958, Z959
Cerebral vascular accident	430~438	I60-166,G450~G452, G458, G459, G46, I64, G454, I69~I672, I674~I679, I681, I682, I688
Dementia	290	F00, F01, F02, F051

© 2021 Huang CY et al. *JAMA Network Open.*

Pulmonary disease	490~496, 500~505	J40~J47, J44, J60-J67
Connective tissue disorder	7100, 7101, 7104, 7140, 7141, 7142, 5171, 725	M32, M34, M332, M053, M058, M059, M060, M063, M069, M050, M052, M051, M353
Peptic ulcer	531~534	K25~K28
Liver disease	5712, 5714, 5715, 5716	K702, K703, K73, K717, K740, K742, K746, K743, K744, K745
Diabetes	2500~2503, 2507	E109, E119, E139, E149, E101, E111, E131, E141, E105, E115, E135, E145
Diabetes complications	2504, 2505, 2506	E102, E112, E132, E142 E103, E113, E133, E143 E104, E114, E134, E144
Paraplegia	342, 3441	G81 G041, G820, G821, G822
Renal disease	582, 5830, 5831, 5832, 5833, 5835, 5836, 5837, 5834, 585, 586, 588	N03, N052~N056, N072, N073, N074, N01, N18, N19, N25
Cancer	14~18, 170, 171, 172, 174, 175, 176, 179, 190~194, 1950~1955, 1958, 200~208	C0~C3, C5, C6, C40~C49, C70~C85, C883, C887, C889, C900, C901, C91~C93, C940, C941, C942, C943, C9451, C947, C95, C96
Metastatic cancer	196~198, 1990, 1991	C77~C80
Severe liver disease	5722~5724, 5728	K729, K766, K767, K721
HIV	042~044	B20~B24

© 2021 Huang CY et al. *JAMA Network Open.*
